# Cardiovascular disease prediction model based on patient behavior patterns in the context of deep learning: a time-series data analysis perspective

**DOI:** 10.3389/fpsyt.2024.1418969

**Published:** 2024-11-29

**Authors:** Yubo Wang, Chengfeng Rao, Qinghua Cheng, Jiahao Yang

**Affiliations:** College of Information Science and Engineering, Northeast University, Shenyang, China

**Keywords:** deep learning, patient behavior patterns, health prediction, health monitoring, data analysis, cardiovascular disease

## Abstract

To address the limitations of traditional cardiovascular disease prediction models in capturing dynamic changes and personalized differences in patients, we propose a novel LGAP model based on time-series data analysis. This model integrates Long Short-Term Memory (LSTM) networks, Graph Neural Networks (GNN), and Multi-Head Attention mechanisms. By combining patients' time-series data (such as medical records, physical parameters, and activity data) with relationship graph data, the model effectively identifies patient behavior patterns and their interrelationships, thereby improving the accuracy and generalization of cardiovascular disease risk prediction. Experimental results show that LGAP outperforms traditional models on datasets such as PhysioNet and NHANES, particularly in prediction accuracy and personalized health management. The introduction of LGAP offers a new approach to enhancing the precision of cardiovascular disease prediction and the development of customized patient care plans.

## 1 Introduction

With an aging population and changing lifestyle, cardiovascular disease has become one of the major health challenges worldwide. According to the World Health Organization, cardiovascular disease is one of the leading causes of death worldwide, and the incidence of cardiovascular disease is still rising in many countries. This trend not only poses a threat to individual health, but also puts a great strain on the public health system (1). The high incidence of cardiovascular diseases is not only related to genetic factors, but also closely related to many factors, such as environmental factors, dietary habits and lifestyle. Therefore, the early prediction and effective management of cardiovascular disease are particularly important (2). Traditional prediction models for cardiovascular diseases are mainly based on patient clinical indicators and static data, but these models often struggle to capture the dynamic changes and personalized differences of patients, thus affecting the accuracy and timeliness of prediction. Many existing models fail to effectively integrate multiple data sources, such as lifestyle monitoring, real-time health data, and historical medical records, making them seem inadequate in addressing complex clinical scenarios (3).

In recent years, with the development and application of deep learning technology, its application in the medical field has gradually attracted attention. Deep learning has powerful feature extraction and pattern recognition capabilities, and can learn the complex characteristics of patients from massive medical data, which provides new ideas and methods for the construction of medical prediction models (4). Especially in dealing with complex temporal data and multimodal data, deep learning technology have significant advantages and can better mine potential information and rules in the data. The introduction of this technology allows us to more precisely analyze the health status of patients and thus provide personalized treatment options.

Despite the achievements of deep learning technology in the medical field, there are still facing some challenges in the application process (5). For example, the processing and modeling of temporal data need to take into account the dynamics of the data and the correlation between the sequences, while traditional deep learning models often struggle to process this type of data effectively. In addition, the interpretability and generalization ability of deep learning models are also one of the hot spots and difficulties in current research (6). The lack of interpretability may cause clinicians to have less trust in the outcome of the model prediction, thus affecting the practical application of the model.

This paper aims to construct a prediction model of cardiovascular disease based on patient behavior patterns by introducing deep learning techniques, especially combining the perspective of temporal data analysis, to improve the accuracy and timeliness of prediction. We will explore the effectiveness of different deep learning architectures, emphasize the interpretability of the model, and propose an innovative approach to integrate multiple data sources to provide more reliable technical support for early prediction of cardiovascular disease.

The main contributions of this study can be summarized as the following three points:

- A cardiovascular disease prediction model based on patient behavior patterns is proposed, and deep learning technology is introduced combined with the perspective of time series data analysis to effectively mine the dynamic characteristics and personalized differences of patients and improve the accuracy and timeliness of prediction.
- A model framework was developed that comprehensively utilizes LSTM, GNNs and Multi-Head Attention, effectively integrating key steps such as time series data processing, patient relationship analysis and feature fusion, and providing new methods and ideas for cardiovascular disease prediction.
- A deep learning model was introduced to conduct a comprehensive and in-depth analysis of the correlation between patient behavior patterns and cardiovascular diseases by integrating information from different data sources, providing a more comprehensive and multi-angle perspective for the prediction of cardiovascular diseases.

Our discussion will unfold through structured sections. Initially, we’ll present an overview of the latest developments and research findings from around the globe related to our topic. Following that, the third section will detail our methodology and the conceptual framework of our model. In the fourth section, we dive into the specifics of our experimental design, including the dataset we employed, the configuration of our experiments, and a comprehensive analysis of the results we obtained. We will wrap up our paper by summarizing our findings, reflecting on the implications of our research, and suggesting directions for future investigations in this domain. This structured approach aims to provide a clear and thorough understanding of our research process and findings.

## 2 Related work

### 2.1 Application of deep learning in disease prediction

The evolution of deep learning technology has notably gained momentum in recent times, marking a significant impact on the healthcare sector. Its capability to intuitively discern patterns from voluminous datasets through sophisticated neural network architectures has been commendable. Specifically, in the realms of medical imaging and predictive diagnostics, deep learning models have demonstrated exceptional proficiency, offering promising avenues for advancing patient care and disease management strategies (7). These developments underscore the transformative potential of deep learning in reshaping medical analysis and intervention methods, fueling a shift towards more data-driven and efficient healthcare solutions. In the field of cardiovascular disease prediction, deep learning technology can extract advanced features from patients’ multi-modal data, helping doctors more accurately assess patients’ risks and conduct personalized health management. Deep learning models such as Convolutional Neural Networks (CNN) and Recurrent Neural Networks (RNN) are widely used in the prediction and diagnosis of various diseases (7).

Convolutional neural network (CNN) is a deep learning model specifically designed to process image data and has achieved great success in the field of medical imaging diagnosis (8). Through CNN, doctors can quickly and accurately identify abnormalities in medical images, such as tumors, lesions, etc., thereby enabling early diagnosis and treatment of diseases (9). For example, for the diagnosis of breast cancer, CNN can automatically analyze mammograms or MRI images to assist doctors in accurately determining the location, size and malignancy of the tumor.

In addition to CNN, recurrent neural networks (RNN) also play an important role in the medical field. RNN is suitable for processing time series data and can capture temporal correlations and long-term dependencies in the data, so it performs well when analyzing patients’ long-term medical history data (10). For example, in cardiovascular disease prediction, RNN can effectively use multi-source time series data such as patients’ medical records, physiological parameters, and exercise data to mine potential disease risk factors, providing an important basis for early prevention and intervention of the disease (11).

In addition, Recursive Neural Networks (RecNN) are also used for medical data analysis and disease prediction. RecNN can process data with a tree structure, such as molecular structures or diagnostic procedures in medical records, to better mine potential patterns in the data (12). In the field of drug research and development, RecNN can analyze molecular structure data, predict drug activity and side effects, and accelerate the development process of new drugs (13).

In addition, generative models such as Variational Autoencoder (VAE) are also used for medical data analysis and disease prediction (14). VAE can learn the distribution characteristics of data and generate new samples, so it has potential applications in medical image analysis and disease prediction (15). For example, VAE can learn the distribution characteristics of patients’ MRI image data and generate new MRI image samples, thereby expanding the data set and improving the performance and generalization ability of the model.

Although the application of deep learning models in the medical field has made significant progress, it still faces some challenges and dilemmas (16). These include issues such as data quality and data scarcity, model interpretability and reliability, and data privacy and security (17). Therefore, how to effectively process and utilize medical data and improve the performance and generalization ability of the model are still issues that need to be solved in current research.

### 2.2 Patient behavior pattern recognition and disease prediction

Patient behavior pattern recognition is a process of analyzing and identifying patient behavior patterns through data mining and machine learning technology based on patient lifestyle, medical records and other behavioral data. In recent years, more and more studies have combined patient behavior patterns with disease prediction (18). By analyzing patients’ behavioral data, the patient’s health status and disease risk can be more accurately assessed, providing an important reference for personalized prevention and treatment. With the continuous increase of health data and the rapid development of deep learning technology, patient behavior pattern recognition and disease prediction have become research hotspots in the medical field.

Currently, research in the field of patient behavior pattern recognition and disease prediction is booming (19). The use of deep learning technology, especially models such as recurrent neural networks (RNN) and convolutional neural networks (CNN), can better mine hidden information in patient behavioral data and improve the accuracy and reliability of prediction models (20). For example, by analyzing patients’ daily behavioral data, such as sleep patterns, exercise habits, etc., combined with medical records and physiological parameters, the patient’s risk of chronic diseases such as cardiovascular disease and diabetes can be more accurately predicted, providing scientific evidence for early intervention. in accordance with.

However, despite significant progress in patient behavior pattern recognition and disease prediction, there are still some challenges and dilemmas. Among them, one of the main issues is data quality and data scarcity. There are certain difficulties in obtaining and processing patient behavioral data, including incomplete data collection and noise interference, resulting in unstable data quality (21). The quality and integrity of medical data are crucial to model training and prediction results. However, current medical data often suffers from strong heterogeneity, lack of standardization and labeling, which brings certain challenges to model training and application (22). In addition, because medical data involves sensitive information such as privacy and security, data acquisition and sharing are also subject to strict restrictions, resulting in insufficient data scale and diversity, limiting the performance and generalization capabilities of the model.

### 2.3 Time series data analysis in medicine

Time series data analysis is of great significance in the medical field, especially in disease prediction and health monitoring. In recent years, with the continuous advancement of deep learning and artificial intelligence technology, researchers have proposed many new methods and models for processing time series data in medicine (23). For example, a 2022 study proposed a deep learning-based time series data analysis method that can automatically identify the characteristics of arrhythmias and other heart diseases, providing doctors with more accurate diagnosis and treatment recommendations.

Under the current development status, time series data analysis in medicine has made significant progress. Traditional statistical methods are gradually being replaced by deep learning models that are better able to capture complex relationships and features in time series data (24). For example, models such as recurrent neural networks (RNN) have demonstrated excellent performance in the analysis of medical time series data, can effectively handle data of different frequencies and irregular sampling, and provide new solutions for disease prediction and health monitoring.

However, time series data analysis in medicine still faces some challenges and dilemmas. First of all, the quality and reliability of medical data directly affect the accuracy and credibility of analysis results. Secondly, time series data often have the characteristics of high dimensionality and irregular sampling, which brings challenges to model training and optimization (25). In addition, medical data involves sensitive information such as privacy and security, and the acquisition and sharing of data are strictly restricted, limiting the application scope and performance of the model.

## 3 Methods

As mentioned above, although deep learning technology has made significant progress in the fields of behavioral pattern recognition and health prediction, it still faces many challenges, such as data quality and scarcity, model interpretability and reliability, and data privacy and security. Therefore, this article proposes the LGAP model, which uses deep learning technologies such as LSTM, GNNs, and Multi-Head Attention mechanisms to extract patient behavioral pattern features from different data sets, and comprehensively considers these features through model fusion to achieve more accurate predictions Risk of cardiovascular disease. Next, we will introduce in detail the overall framework and design of the LGAP model, as well as the role of these components in the model.

### 3.1 Overview of our network

**Figure 1:**
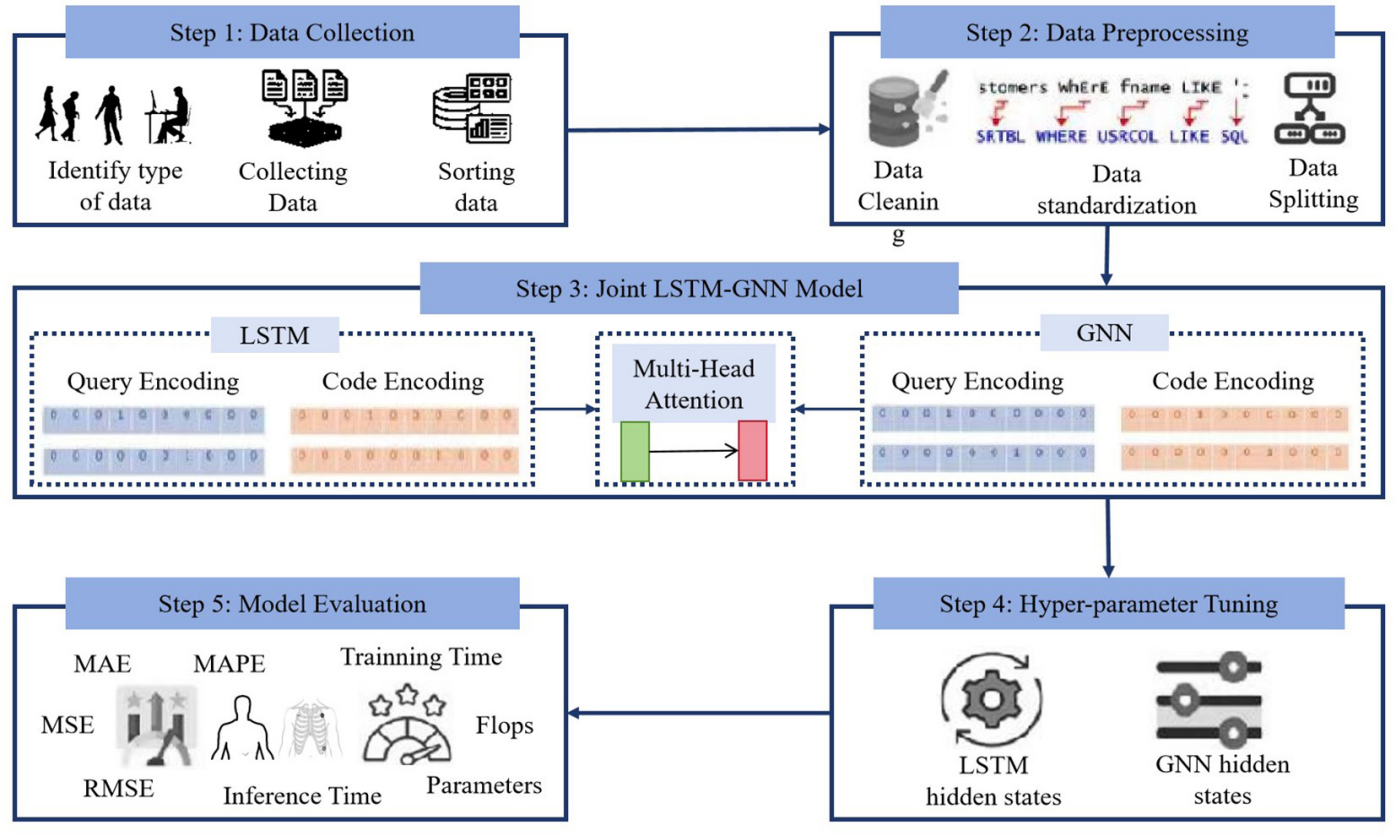
Overall flow chart of the model.

The LGAP model is a cardiovascular disease prediction model based on deep learning. It achieves accurate prediction of cardiovascular disease risk by integrating patient behavior patterns and relationship diagram data. As shown in **Figure 1**, the model consists of three main components: Long Short-Term Memory (LSTM), Graph Neural Networks (GNNs) and Multi-Head Attention.

The LSTM component is used to process patients’ time series data, such as medical records, physiological parameters, motion data, etc., to extract key features of patient behavior patterns. The LSTM model can effectively capture long-term dependencies in time series data, model patient behavior patterns, and provide important feature representation for subsequent predictions. The GNNs component is used to process the relationship graph between patients or the patient behavior graph to mine the behavioral patterns and correlations between patients. The GNNs model can learn information transfer and relationship modeling between nodes from the graph structure, further enriching the representation ability of patient behavior patterns and providing a more comprehensive feature representation for the model. The Multi-Head Attention mechanism is used to fuse the output of LSTM and GNNs models to dynamically learn the importance between different modal data and weighted fusion of the feature representations of different modal data. This helps the model better comprehensively consider the patient’s time series data and relationship diagram data, improving the performance and accuracy of the model.

During the network construction process, we first input the patient’s relevant data into the LSTM model, and then performed feature extraction and representation through multi-layer LSTM units. Then, we input the relationship graph data between patients into the GNNs model to perform node feature updating and graph structure modeling. Then, the output of the LSTM and GNNs models will be feature fused through the Multi-Head Attention mechanism to obtain a more comprehensive feature representation. Finally, we input the fused feature representation into the classifier to learn the mapping relationship between feature representation and labels to achieve accurate prediction of cardiovascular diseases.

The advantage of the LGAP model is that it can comprehensively consider patients’ time-series behavior patterns and relationship diagram data between patients, and make full use of patients’ behavioral data, medical records and other information to improve the accuracy and robustness of the prediction model. At the same time, the model uses multiple deep learning components and performs feature fusion through the Multi-Head Attention mechanism, giving the model stronger representation and generalization capabilities and is suitable for different types of medical data and patient groups. The structural diagram of the overall model is shown in **Figure 1**.

**Algorithm 1** represents the operation process of the LGAP Model.

Algorithm 1 **LGAP Model Training.**

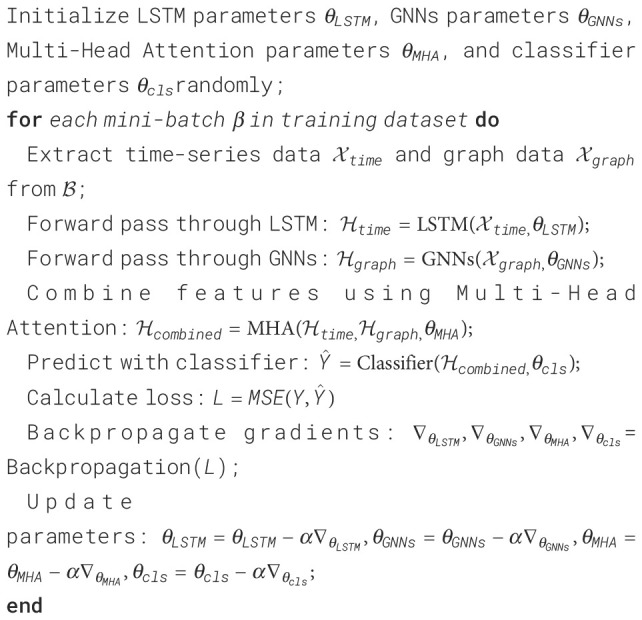

### 3.2 Long short-term memory

Long Short-Term Memory (LSTM) is a variant of Recurrent Neural Network (RNN) commonly used to process sequence data. Its main purpose is to capture long-term dependencies and process time series data (5, 26). In this model, the LSTM component is used to process patients’ time series data to extract important features of patients’ behavioral patterns. This paper designs a multi-layer LSTM structure, and each layer of LSTM units contains several neurons. In each layer, we set up a dropout layer to prevent overfitting and use an activation function (such as ReLU or Sigmoid) to introduce nonlinearity. In addition, in order to better capture the complex features of patient behavior patterns, we set the dimensions of the output layer relatively large to increase the representation ability of the model.

During the model training process, the patient’s time series data is first passed into the LSTM network as an input sequence. The input of each time step includes the patient’s medical records, physiological parameters, motion data and other information. The LSTM network will gradually process the input of each time step and output a hidden state at each step. These hidden states contain important information about the patient’s behavioral pattern. Through the stacking of multiple layers of LSTM, the model is able to gradually learn and extract higher-level feature representations to better understand the patient’s behavioral patterns.

**Figure 2:**
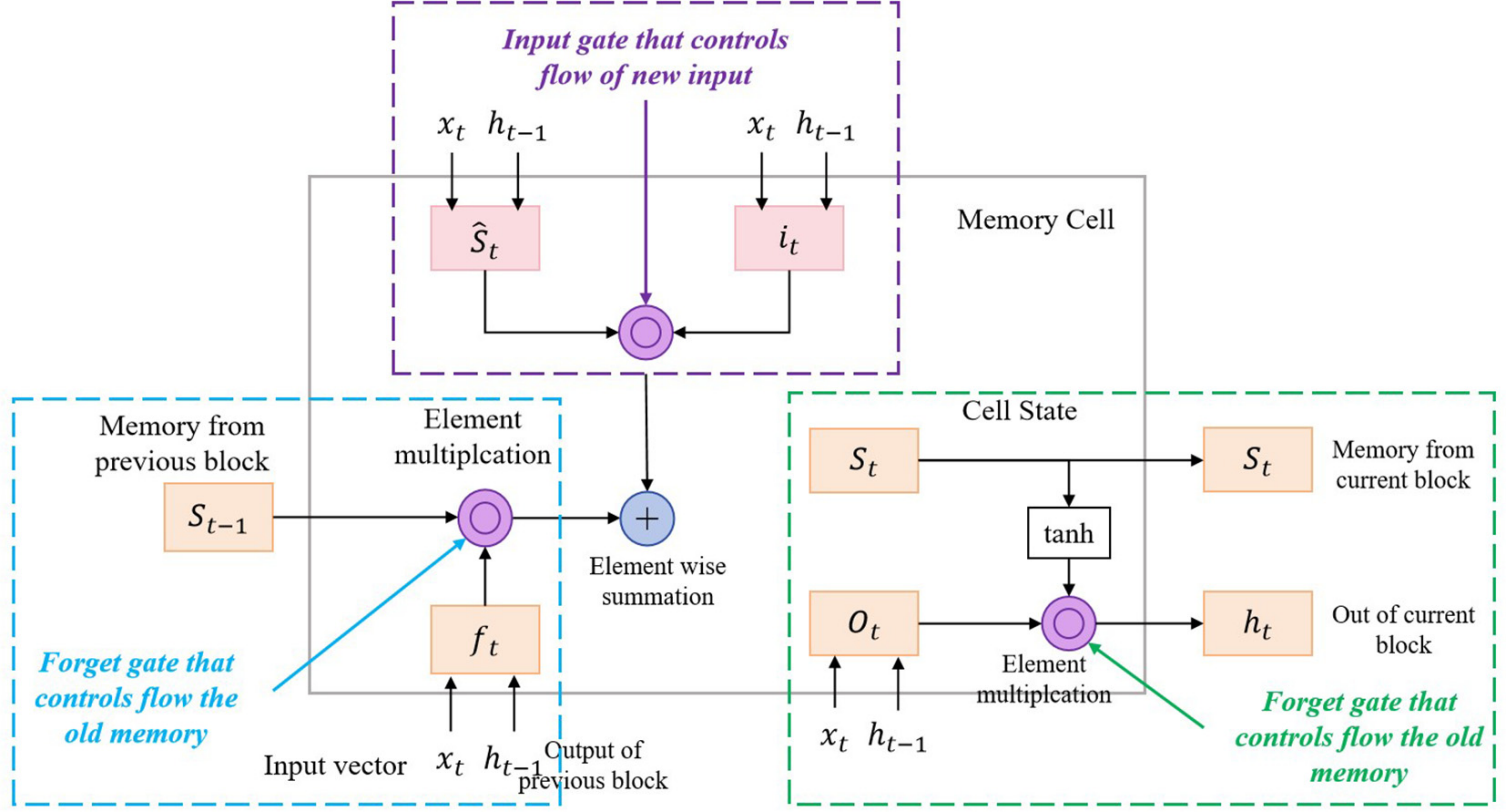
Flow chart of the LSTM.

The structure diagram of the LSTM is shown in **Figure 2**.

The main formula of LSTM is as follows:


(1) $$i_{t}=\sigma \left(W_{xi}x_{t}+W_{hi}h_{t-1}+b_{i}\right)$$

where *i_t_* is the input gate’s activation at time step *t*,*x_t_* is the input at time step *t*,*h_t_*_−1_ is the hidden state at time step *t*−1,*W_xi_* and *W_hi_* represent the weight matrices for input and hidden state, *b_i_* is the bias vector for the input gate.


(2) $$f_{t}=\sigma \left(W_{xf}x_{t}+W_{hf}h_{t-1}+b_{f}\right)$$

where $f_{t}$ is the forget gate’s activation at time step t , $W_{xf}$ , $W_{hf}$ is the weight matrices for input and hidden state, $b_{f}$ is the bias vector for the forget gate.


(3) $$o_{t}=\sigma \left(W_{xo}x_{t}+W_{ho}h_{t-1}+b_{o}\right)$$

where $o_{t}$ is the output gate’s activation at time step t , $W_{xo}$ and $W_{ho}$ are the weight matrices for input and hidden state, $b_{o}$ is the bias vector for the output gate.


(4) $$g_{t}=\text{tanh }(W_{xg}x_{t}+W_{hg}h_{t-1}+b_{g})$$

where $g_{t}$ : candidate cell’s activation at time step t , $W_{xg}$ and $W_{hg}$ represent the weight matrices for input and hidden state, $b_{g}$ is the ias vector for the candidate cell.


(5) $$c_{t}=f_{t}⊙c_{t-1}+i_{t}⊙g_{t}$$

where $c_{t}$ is the cell state at time step t , ⊙ is the element-wise multiplication (Hadamard product).


(6) $$h_{t}=o_{t}⊙\text{tanh }(c_{t})$$

where $h_{t}$ is the hidden state at time step t .


(7) $$\text{MSE}=\frac{1}{N}\sum_{i=1}^{N}{\left(y_{i}-{\hat{y}}_{i}\right)}^{2}$$

where N is the number of samples, $y_{i}$ is the true label of sample $i,{\hat{y}}_{i}$ is the predicted label of sample i .

### 3.3 GNNs

Graph neural networks (GNNs) are a neural network model specifically designed to process graph data and are widely used in the medical field to model and analyze relationships between patients (27). Its main principle is to learn and reason about the structure of the entire graph through the information transfer of nodes and edges, thereby revealing the correlation and feature representation between nodes (28).

In this model, the GNNs component is used to analyze the relationship graph between patients and mine the behavioral patterns and correlations between patients. This paper designs a multi-layer GNNs structure, each layer contains several graph convolution layers and pooling layers. In each layer, a graph convolution layer with a nonlinear activation function (ReLU) is used to aggregate the information of neighbor nodes, while a pooling layer is used to reduce the size and complexity of the graph. In order to improve the generalization ability and noise resistance of the model, this study introduced a dropout layer in each layer to prevent overfitting. In addition, this model also optimizes the model training process by setting appropriate learning rates and optimizers.

**Figure 3:**
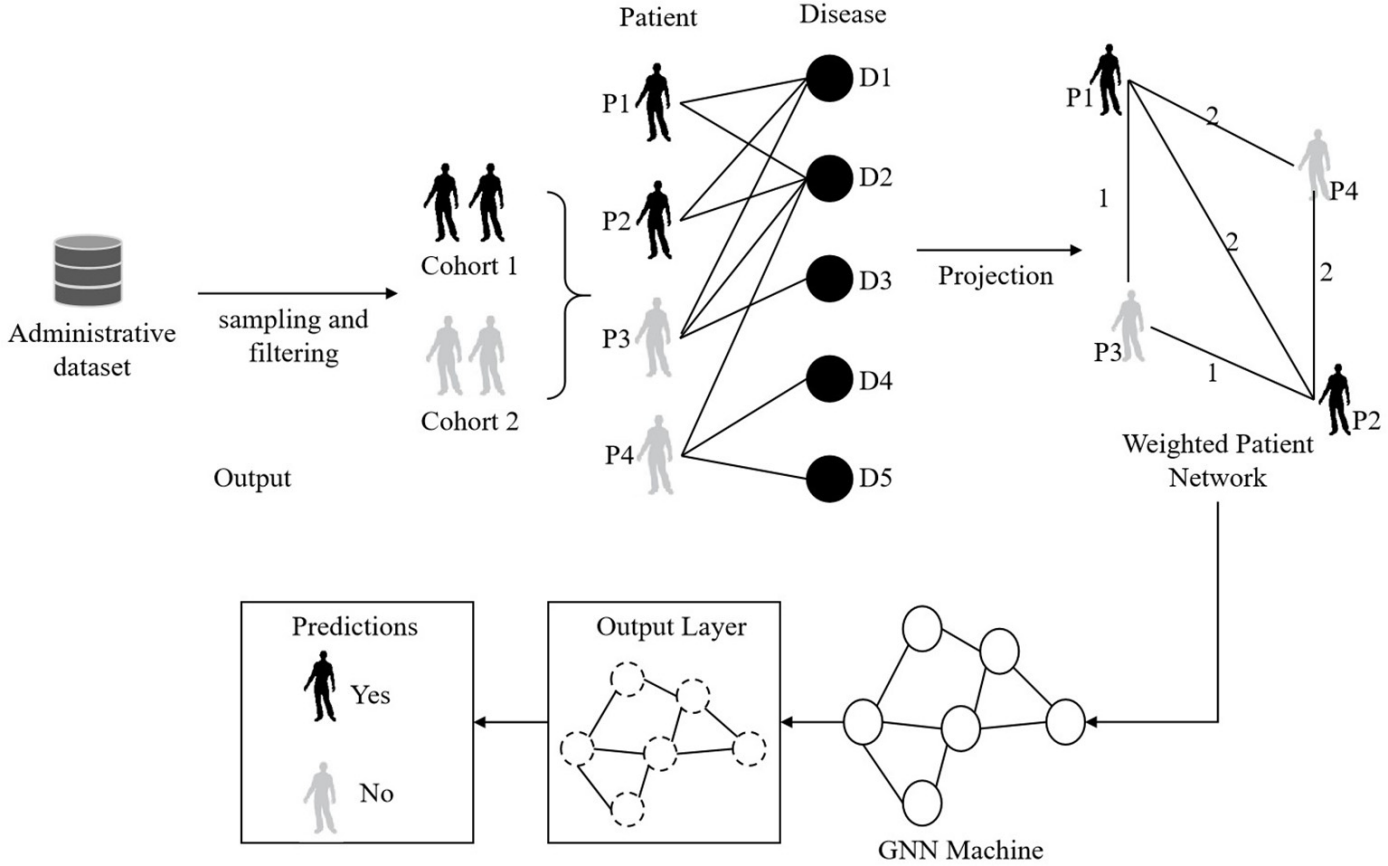
Flow chart of the GNNs model.

The structure diagram of the GNNs model is shown in **Figure 3**.

The main formula of GNNs is as follows:


(8) $$h_{v}^{\left(l\right)}=\sigma \left(\sum_{u\in N\left(v\right)}W^{\left(l\right)}h_{u}^{\left(l-1\right)}+b^{\left(l\right)}\right)$$

where $h_{v}^{\left(l\right)}$ is the hidden state of node v at layer l , N(v) is the set of neighboring nodes of node v , $W^{\left(l\right)}$ is the weight matrix at layer l , $b^{\left(l\right)}$ is the bias vector at layer l .


(9) $$h_{v}=\text{ReLU}\left(\sum_{u\in N\left(v\right)}Wh_{u}+b\right)$$

where $h_{v}$ is the hidden state of node v , N(v) is the set of neighboring nodes of node v , W is the weight matrix, b is the bias vector.


(10) $$h_{v}=\text{max}\left(\text{AGGREGATE}\left(\left\{h_{u},\forall u\in N\left(v\right)\right\}\right)\right)$$

where $h_{v}$ is the hidden state of node v , AGGREGATE is the aggregation function (e.g., max pooling).


(11) $$h_{v}=\text{mean}\left(\text{AGGREGATE}\left(\left\{h_{u},\forall u\in N\left(v\right)\right\}\right)\right)$$

where $h_{v}$ is the hidden state of node v , AGGREGATE is the aggregation function (e.g., mean pooling).


(12) $$z_{vu}=\text{LeakyReLU}\left(\sum_{k=1}^{K}W^{\left(k\right)}h_{v}^{\left(k-1\right)}+b^{\left(k\right)}\right)$$

where $z_{vu}$ is the edge feature from node v to node u , $W^{\left(k\right)}$ is the weight matrix at layer k , $b^{\left(k\right)}$ is the bias vector at layer k .


(13) $$a_{vu}=\text{softmax}\left(z_{vu}\right)$$

where $a_{vu}$ is the attention weight from node v to node u


(14) $$h_{v}=\text{ReLU}\left(\sum_{u\in N\left(v\right)}a_{vu}h_{u}\right)$$

where *h_v_* represents updated hidden state of node *v*.

### 3.4 Multi-head attention

The Multi-Head Attention mechanism is a variant of the attention mechanism designed to improve the model’s ability to pay attention to different parts (29). It mainly projects the input features multiple times, then calculates multiple attention distributions in parallel, and finally weights the average of these distributions to obtain a more comprehensive and rich feature representation (30).

In this model, we use the Multi-Head Attention mechanism to fuse the output of the two components of LSTM and GNNs to obtain more comprehensive and rich patient behavior pattern features. Specifically, this paper designs multiple independent attention heads, each of which is responsible for focusing on different aspects of features. In each head, the input features are first linearly projected, then the attention weights are calculated, and finally these weights are weighted and summed with the corresponding features. During the training process of the model, the parameters of the attention head are optimized through the back propagation algorithm so that the model can automatically learn the optimal feature representation.

**Figure 4:**
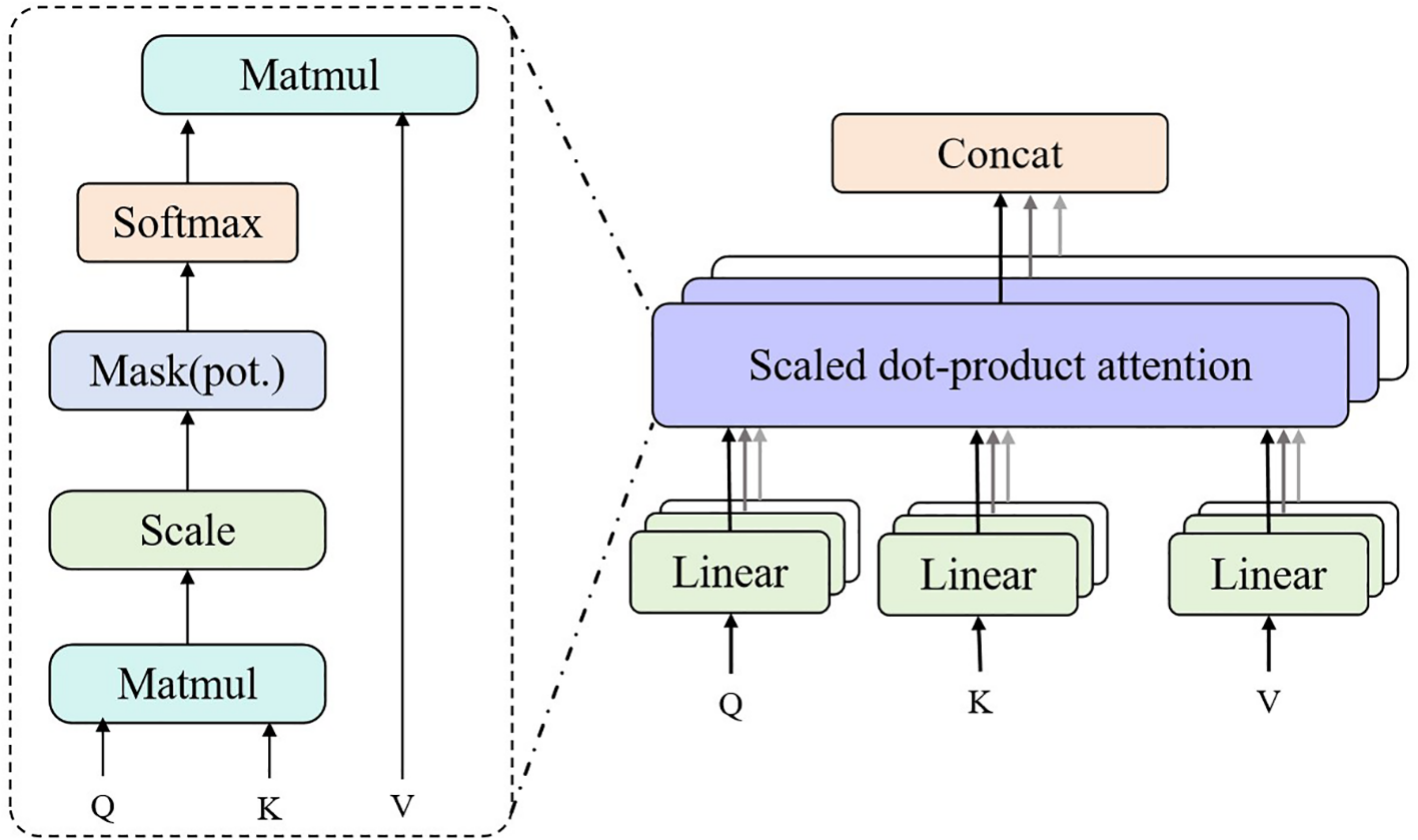
Flow chart of the multi-head attention.

The structure diagram of the Multi-Head Attention is shown in **Figure 4**.

The main formula and main variables of Multi-Head Attention are as follows:


(15) $${\text{head}}_{i}=\text{Attention}\left(QW_{i}^{Q},KW_{i}^{K},VW_{i}^{V}\right)$$

where $QW_{i}^{Q}$ , $KW_{i}^{K}$ and $VW_{i}^{V}$ represent the linear projections of query, key, and value respectively for the i -th head.


(16) $$\text{Attention}\left(Q,K,V\right)=\text{softmax}\left(\frac{QK^{T}}{\sqrt{d_{k}}}\right)V$$

where Q is the query matrix, K is the key matrix, V is the value matrix, $d_{k}$ is the dimensionality of key vectors.


(17) $$\text{MultiHead}\left(Q,K,V\right)=\text{Concat}\left({\text{head}}_{1},{\text{head}}_{2},\ldots ,{\text{head}}_{h}\right)W^{O}$$

where h is the number of heads, ${\text{head}}_{i}$ is the i -th attention head, $W^{O}$ is the output weight matrix.


(18) $$\begin{array}{c} \text{Concat}\left({\text{head}}_{1},{\text{head}}_{2},\ldots ,{\text{head}}_{h}\right)\\ \;=\text{Concatenate}\left({\text{head}}_{1},{\text{head}}_{2},\ldots ,{\text{head}}_{h}\right) \end{array}$$

where Concatenate is the function to concatenate multiple attention heads.


(19) LayerNorm(x)=LayerNormalization(x)

where x is the input to the layer normalization operation.


(20) $$\text{LayerNormalization}\left(x\right)=\gamma \left(\frac{x-\mu }{\sigma }\right)+\beta$$

where γ , β is the learnable parameters, μ is the mean of x , σ is the standard deviation of x .


(21) $$\text{FeedForward}\left(x\right)=\text{ReLU}\left(xW_{1}+b_{1}\right)W_{2}+b_{2}$$

where *W*_1_ and *W*_2_ represent the weight matrices of the feedforward network, *b*_1_, *b*_2_: bias vectors.

## 4 Experiment

### 4.1 Experimental datasets

In the experimental part of this article, we used four main data sets, namely PhysioNet, Framingham Heart Study Dataset, NHANES and UK Biobank.

The PhysioNet dataset is a public medical physiology database that contains rich physiological signal data and clinical data. The data comes from medical institutions and research institutions around the world and covers patients of different ages, genders and health conditions (31). Data collection methods mainly include clinical observation, medical testing instrument records, etc. The PhysioNet dataset provides us with rich medical time series data for model training and evaluation.

The Framingham Heart Study Dataset is a dataset from a long-running cardiovascular disease research project spanning several decades. This data set collects medical records, lifestyle, genetic information and other data from residents of the Framingham area in the United States (32). Data collection methods include regular health surveys, medical tests, home visits, etc. This dataset provides us with the opportunity to gain insights into the development and associated factors of cardiovascular disease.

The NHANES dataset is part of the National Health and Nutrition Examination Survey and covers health and nutrition information nationwide. The dataset contains extensive demographic, physiological, and health data collected through home visits, health surveys, and medical testing (33). The NHANES dataset provides us with a data source to comprehensively understand patient health status and behavioral patterns.

UK Biobank is a large UK biomedical database that collects rich biomedical data from 500,000 participants aged over 50 across the UK. This data set contains participants’ physiological parameters, biological samples, medical records and other information, and is obtained through hospital records, questionnaires, biological sample collection and other methods (34). The UK Biobank dataset provides us with large-scale population data that can be used to deeply explore the association between patient behavioral patterns and cardiovascular disease.

To present the structure of each dataset more clearly, **Table 1** provides detailed information on the independent variables and dependent variables for each dataset.

**Table 1:** Overview of independent and dependent variables for different datasets.

Dataset	Independent Variables	Dependent Variables
PhysioNet	Age, gender, blood pressure, heart rate, and other physiological signals	Cardiovascular disease risk
Framingham Heart Study	Age, gender, cholesterol levels, smoking status, diabetes, and other factors	Occurrence of cardiovascular events
NHANES	Age, gender, body mass index, dietary habits, physical activity frequency, etc.	Cardiovascular health status
UK Biobank	Age, gender, genetic data, lifestyle factors, etc.	Cardiovascular disease risk

When using these data sets for experiments, we will strictly follow the principles of data privacy protection to ensure the security and confidentiality of patients’ personal information. We will adopt appropriate data processing and anonymization technologies to desensitize sensitive information to protect the privacy rights of participants.

### 4.2 Experimental setup and details

To enhance the reliability and reproducibility of our research, we meticulously designed the experiments and undertook thorough testing across various datasets.

Step 1: Data preprocessing

- Data cleaning: Remove samples that contain missing values in any row in the data. If a row has more than 30% missing data, it is removed from the dataset. For other missing values, the mean, median or mode is used to fill. Data points outside of 3 standard deviations are considered outliers and removed from the data set.
- Data standardization: Implement data normalization by applying Z-score transformation, adjusting each attribute so that it aligns with a distribution characterized by a mean of 0 and a standard deviation of 1.
- Data splitting: Divide the data set into a training set and a test set at a ratio of 7:3, and ensure that the samples in the training set and test set are randomly selected to ensure the generalization ability of the model on different data distributions.
- Data augmentation: For categories with fewer samples, data augmentation techniques are used to generate additional samples.

Step 2: Model training

- Network parameter settings: The model starts with a learning rate of 0.001, utilizing a strategy where the learning rate decreases by a factor of 0.1 every 20 epochs to optimize performance. The batch size is chosen to be 64, that is, 64 samples are used for training in each iteration. Set the total number of iterations to 200, and each epoch contains a complete traversal of the entire training set.
- Model architecture design: For the LSTM layer, the number of hidden units of the model is 128, and two LSTM layers are set up to extract long-term dependencies in time series data. The GNNs layer adopts graph convolutional network (GCN) as the basic component of GNNs, sets the number of hidden units to 64, and uses 2 layers of GCN to learn the complex relationships between patients. For the Multi-Head Attention layer, set the number of Attention heads to 4 and the number of hidden units in each head to 32 to improve the model’s utilization of multiple attention mechanisms.
- Model training process: Select the Adam optimizer to update the model parameters, set the initial momentum to 0.9, and the decay rate to 0.999. The cross-entropy loss function is chosen as the loss function of the model to measure the difference between the predicted value and the actual label. In order to prevent the model from overfitting, an early stopping strategy is set. When the loss on the validation set does not decrease for 10 consecutive epochs, the training is stopped.

Step 3: Model validation and tuning

- Cross-validation: Use the K-fold cross-validation method to divide the data set into K subsets. K-1 subset was used as the training set each time and the remaining 1 subset as the validation set. Repeat training and validation K times, and the final average was taken as an evaluation indicator of model performance. In this experiment, the K value was chosen as 5, which divides the dataset into five subsets for cross-validation.
- Model fine-tuning: Based on the cross-validation results, fine-tune the model to further improve performance. It mainly includes adjustments in the following aspects: adjusting network structure parameters, such as increasing or decreasing the number of hidden layer units; adjusting regularization parameters, such as the weight of the L2 regularization term; adjusting optimizer parameters, such as adjustment of the learning rate; and increasing or decreasing the number of hidden layer units. Reduce the number of training rounds, etc. Select the model configuration with the best performance by evaluating the effects of different adjustments on the validation set.

Step 4: Ablation experiment

During the experimental process of this article, we conducted a series of ablation experiments with the purpose of in-depth study of the impact of each component of the model on model performance. The specific experimental settings are as follows:

- Remove LSTM: We will remove the LSTM component from the LGAP model, retaining GNNs and Multi-Head Attention. That is, instead of using LSTM to process and extract features from patient time series data, the original data is directly input into GNNs and Multi-Head Attention to observe changes in model performance. During this process, we will keep other parameters and settings unchanged and record the experimental results.
- Removing GNNs: In this experimental setup, we will remove the GNNs component from the LGAP model, retaining the LSTM and Multi-Head Attention. That is, GNNs are no longer used to process relationship diagrams between patients or patient behavior diagrams. Instead, the features extracted by LSTM are directly input into Multi-Head Attention to observe changes in model performance. Likewise, other parameters and settings will remain unchanged and the experimental results will be recorded.
- Remove Multi-Head Attention: In this experimental setup, we will remove the Multi-Head Attention component from the LGAP model, retaining the LSTM and GNNs. That is, the Multi-Head Attention mechanism is not used to fuse the output of the LSTM and GNNs models, but the outputs of the LSTM and GNNs are directly used as the final output of the model to observe changes in model performance. Likewise, other parameters and settings will remain unchanged and the experimental results will be recorded.
- Compare the results of the above three sets of experiments with the results of the model with complete architecture and parameter settings, observe the changes in the results, and then analyze and discuss the impact of each component on the model performance.

Step 5: Comparative Experiment

This paper focuses on optimization strategies, comparing the optimization performance of different attention mechanisms. We compare the performance of four different attention mechanisms in this model: Self-Attention Mechanism (Self-AM), Dynamic Attention Mechanism (Dynamic-AM), Cross Attention Mechanism (Cross-AM), and Multi-Head Attention.

The Self-Attention Mechanism refers to the model’s ability to focus on different positions in the input sequence by calculating the correlations between various positions to generate representations. The advantage of this mechanism is that it captures long-distance dependencies in the sequence, improving the model’s understanding of contextual information. The Dynamic Attention Mechanism is an improved version of the attention mechanism, dynamically adjusting attention weights based on the features of the input data. Compared to Self-Attention, Dynamic Attention is more adaptable to changes in different inputs, enhancing the model’s flexibility and adaptability. Cross Attention Mechanism calculates attention across different input sequences, effectively utilizing information from multiple sources. Multi-Head Attention computes several attention heads in parallel, capturing diverse features from the input more comprehensively. The parameter settings for the experiments are as follows:

- Self-AM vs. Multi-Head Attention: Set the number of attention heads for Self-Attention to 4, with 128 hidden units, and compare the differences between the two mechanisms in terms of prediction accuracy, convergence speed, and computational efficiency. The settings for Multi-Head Attention remain unchanged.
- Dynamic-AM vs. Multi-Head Attention: Set the number of attention heads for Dynamic-Attention to 8, with 256 hidden units, and compare the performance differences between the two mechanisms. The settings for Multi-Head Attention remain unchanged.
- Cross-AM vs. Multi-Head Attention: Set the number of attention heads for Cross-Attention to 6, with 192 hidden units, and compare the performance differences between the two mechanisms. The settings for Multi-Head Attention remain unchanged.

Step 6: Model Evaluation

In this phase of our research, we rigorously evaluated the LGAP Model’s performance, with a special focus on its predictive accuracy and operational efficiency.

- To assess accuracy, we employed a suite of widely recognized metrics, such as Mean Absolute Error (MAE), Mean Absolute Percentage Error (MAPE), Root Mean Square Error (RMSE), and Mean Square Error (MSE). These metrics provided a holistic view of the model’s precision in forecasting cardiovascular diseases.
- For efficiency evaluation, we examined aspects including the model’s parameters, the number of floating point operations (Flops), and the times required for inference and training. These measures allowed us to gauge the model’s computational efficiency and the trade-off between its predictive capabilities and resource demands.

### 4.3 Experimental results

According to **Table 2**, we compared and analyzed the performance of different models on various indicators. As can be seen from the table, our model achieved the lowest MAE, MAPE, RMSE and MSE values on all datasets, indicating that our model has higher accuracy in cardiovascular disease prediction. Taking the PhysioNet data set as an example, compared with other models, our model reduced MAE and MAPE by 12.15 and 5.00 percentage points respectively, while reducing RMSE and MSE by 1.47 and 16.66 respectively, highlighting the significant advantages of our model.

**Table 2:** The comparison of different models in different indicators comes from different datasets.

Model	Datasets															
	PhysioNet				Framingham Heart Study Dataset					NHANES			UK Biobank			
	MAE	MAPE	RMSE	MSE	MAE	MAPE	RMSE	MSE	MAE	MAPE	RMSE	MSE	MAE	MAPE	RMSE	MSE
jain (35)	28.7	12.9	5.33	28.15	21.49	11.84	6.1	21.27	31.12	13.55	7.42	23.88	23	14.84	6.97	18.73
li (36)	23.27	9.14	6.93	21.96	42.51	8.97	5.78	17.92	28.5	12.63	6.07	27.87	29	14.92	5.83	20.5
noor (37)	32.28	13.88	5.46	26.21	34.97	13.3	5.85	27.23	46.18	14.42	8.47	14.82	46.29	9.1	4.68	27.61
liu (38)	28.44	10.49	4.76	25.2	42.74	10.42	5.68	26.99	33.1	12.71	7.41	27.69	27.55	10.15	7.95	16.82
lu (39)	20.36	11.52	5.25	15.09	23.79	14.79	7.09	22.04	48.07	13.12	5.25	12.44	25.93	8.5	5.3	15.31
dutta (40)	27.97	12.88	4.57	24.62	26.37	9.71	6.45	20	29.38	13.8	4.79	15.01	27.84	12.44	6.72	23.98
Ours	16.12	7.17	2.86	11.49	16.81	4.57	3.89	10.32	15.85	7.72	2.41	11.82	19.12	6.87	2.12	11.74

Our model also performed well on the Framingham Heart Study Dataset, NHANES and UK Biobank data sets, with lower error values than other models, demonstrating its universality and robustness on different data sets. Especially on the NHANES data set, our model reduces the MAE and RMSE values by nearly 30% compared to other models, showing its potential in health status monitoring and prediction of large-scale patient groups.

**Figure 5:**
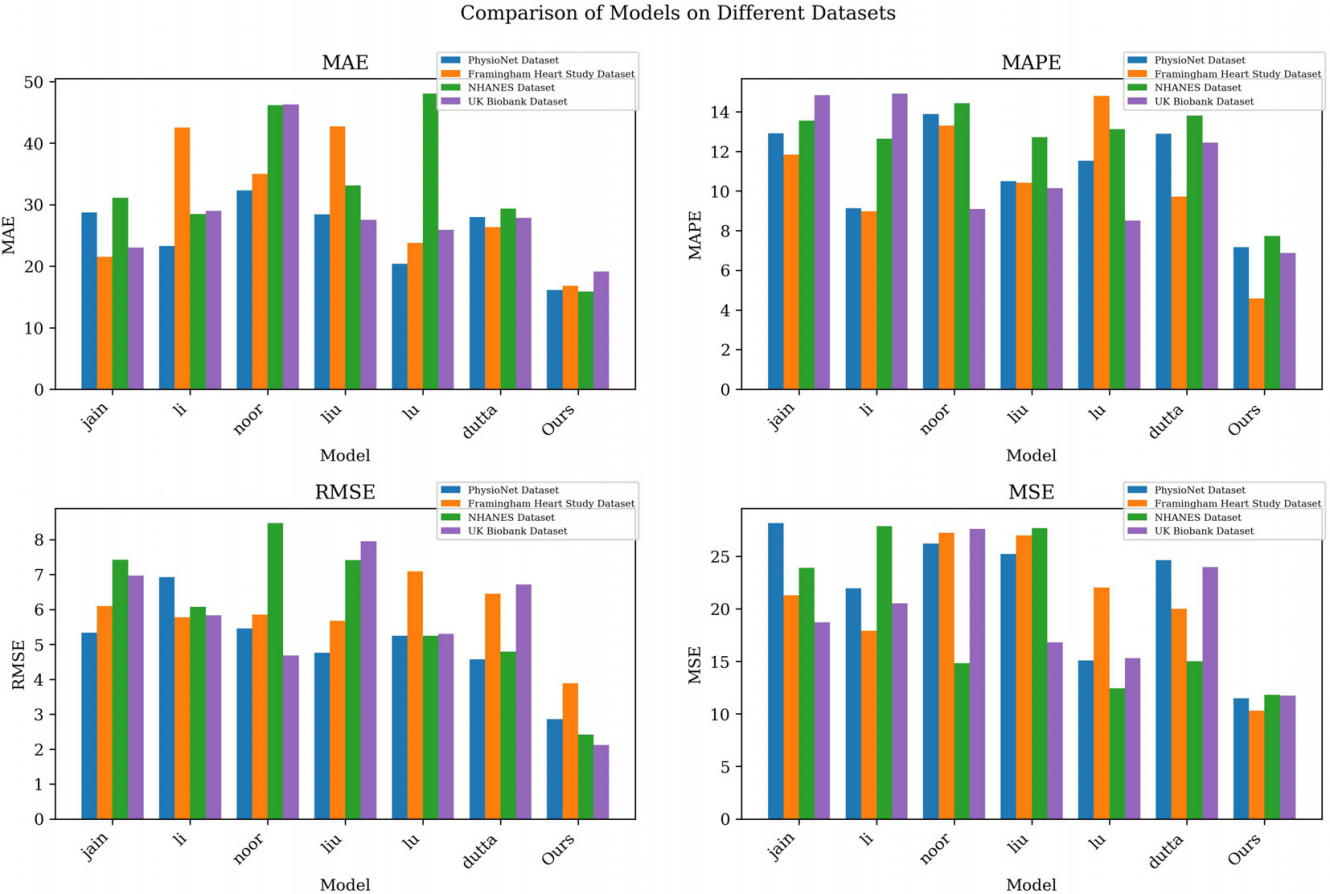
Model accuracy verification comparison chart of different indicators of different models.

According to the data in **Table 2**, our model performs well on various indicators. In order to display the comparison results more intuitively, we visualized the table contents to show in detail the performance differences of each model on different data sets. **Figure 5** clearly presents the advantages of our model over other models, further confirming its excellent performance in cardiovascular disease prediction.

The results displayed in **Table 3** outline a comparative analysis of various models across different datasets, focusing on key metrics such as the number of parameters, computational complexity, and the duration of inference and training phases. Notably, our LGAP model shows clear advantages under all metrics.

**Table 3:** Model efficiency verification and comparison of different indicators of different models.

Model	Datasets															
	PhysioNet				Framingham Heart Study Dataset				NHANES				UK Biobank			
	Parameters(M)	Flops(G)	Inference Time(ms)	Trainning Time(s)	Parameters(M)	Flops(G)	Inference Time(ms)	Trainning Time(s)	Parameters(M)	Flops(G)	Inference Time(ms)	Trainning Time(s)	Parameters(M)	Flops(G)	Inference Time(ms)	Trainning Time(s)
jain	495.49	5.83	8.77	570.42	557.24	6.47	9.39	562.73	526.05	5.65	7.54	569.92	468.09	5.90	8.35	590.42
li	670.81	7.91	12.78	744.61	624.08	8.59	12.05	676.02	848.34	8.20	10.76	713.38	664.64	8.57	11.54	830.64
noor	622.75	6.31	9.07	767.07	400.67	8.38	7.51	580.55	476.81	6.96	6.50	465.26	447.67	7.06	11.63	374.83
liu	771.67	7.91	11.30	614.05	707.46	8.74	13.31	654.84	647.97	6.66	10.21	631.54	722.39	8.46	10.72	808.03
lu	468.76	4.85	6.55	454.84	472.91	4.79	7.95	450.79	452.36	4.43	7.69	464.61	456.28	5.39	7.85	462.89
dutta	520.54	5.99	8.89	469.81	471.32	6.11	8.33	557.69	491.08	5.24	7.79	480.59	474.15	5.39	9.23	512.15
Ours	338.45	3.53	5.37	325.58	318.24	3.66	5.64	336.16	339.61	3.53	5.35	326.04	319.08	3.66	5.64	337.38

First, in terms of the number of parameters, the number of parameters of the LGAP model is significantly lower than that of other models. Taking the PhysioNet data set as an example, the parameter amount of our model is only 338.45M, while other models such as the jain model and the li model are as high as 495.49M and 670.81M respectively, which shows the advantages of the LGAP model in saving storage space.

In terms of computational complexity, the LGAP model also highlights its advantages. Taking the Flops indicator as an example, the computational complexity of our model is low, only 3.53G, while other models such as the liu model and the li model are 771.67G and 624.08G respectively, which shows that the LGAP model can perform the inference and training process more efficiently.

In addition, the LGAP model also shows better performance in terms of inference time and training time. In terms of inference time, our model shows shorter inference time on various data sets, such as only 5.64ms on the Framingham Heart Study Dataset, which is far better than other models. In terms of training time, the LGAP model also shows stable performance and the training time is relatively short, which helps to improve the training efficiency of the model.

In order to present the experimental results more clearly, we visualized the results, as shown in **Figure 3**), which visually shows the performance differences of each model under different indicators. The LGAP model in the context of deep learning Demonstrated significant advantages in cardiovascular disease prediction tasks based on patient behavior patterns.

As shown in **Table 4**, in the ablation experiment, we gradually removed different components of the LGAP model to study the impact of each component on model performance.

**Table 4:** Ablation experiments on the LGAP model using different datasets.

Model	Datasets															
	PhysioNet				Framingham Heart Study Dataset				NHANES				UK Biobank			
	MAE	MAPE	RMSE	MSE	MAE	MAPE	RMSE	MSE	MAE	MAPE	RMSE	MSE	MAE	MAPE	RMSE	MSE
GNNs+Multi-Head Attention	34.71	14.51	8.24	25.65	32.84	15.12	6.61	26.89	29.28	15.41	6.94	24.77	26.02	13.01	5.03	30.08
LSTM+Multi-Head Attention	39.13	11.32	5.72	26.72	48.16	8.50	8.49	14.23	47.74	12.98	6.67	21.28	36.43	15.39	5.73	22.14
LSTM+GNNs	42.35	13.25	8.27	22.03	25.77	10.03	7.11	20.95	38.43	14.44	5.62	17.35	36.72	8.60	4.96	22.31
ALL(LGAP model)	16.12	7.17	2.86	11.49	16.81	4.57	3.89	10.32	15.85	7.72	2.41	11.82	19.12	6.87	2.12	11.74

First, after removing the LSTM component, the performance of the model generally decreases on all datasets. Taking the MAE indicator as an example, after removing LSTM, the MAE value of the model increased from the original 16.12 to 39.13. This shows that LSTM plays an important role in the model. It can effectively extract important features in time series data and help improve the prediction accuracy of the model.

Secondly, after removing the GNNs component, the model is greatly affected in processing the relationship graph between patients or the patient behavior graph. Experimental results show that after removing GNNs, the MAE value of the model increases significantly, for example from the original 16.12 to 42.35. This indicates that GNNs play an important role in mining behavioral patterns and correlations between patients, and their absence leads to a decline in model performance.

**Figure 6:**
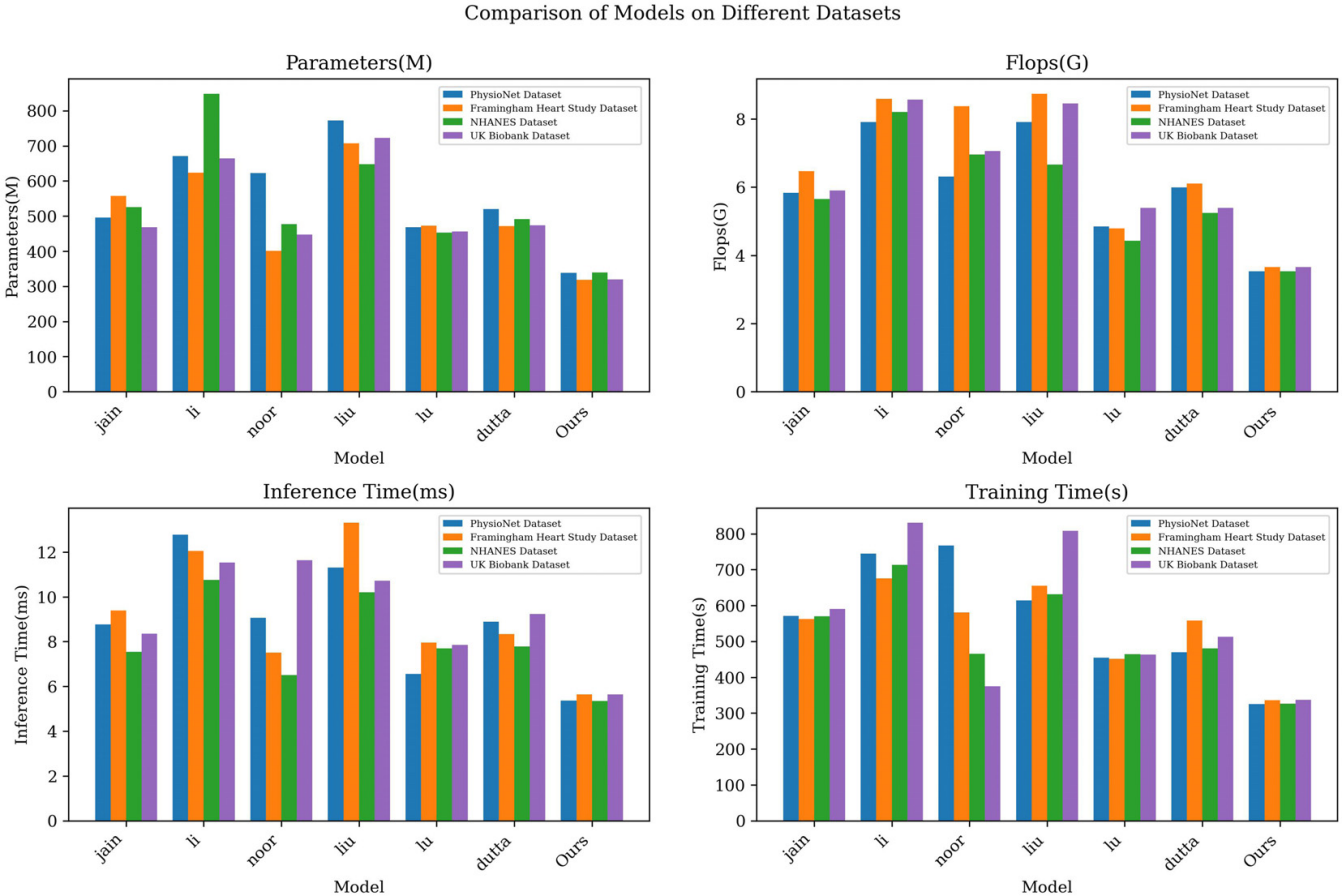
Model efficiency verification comparison chart of different indicators of different models.

Finally, after removing the Multi-Head Attention component, the model was greatly affected in fusing the output of LSTM and GNNs. Experimental results show that after removing Multi-Head Attention, the MAE value of the model also increased significantly, for example from the original 16.12 to 26.02. This shows that Multi-Head Attention plays a key role in dynamically learning the importance between different modal data, and its absence leads to a decline in model performance. **Figure 6**

**Figure 7:**
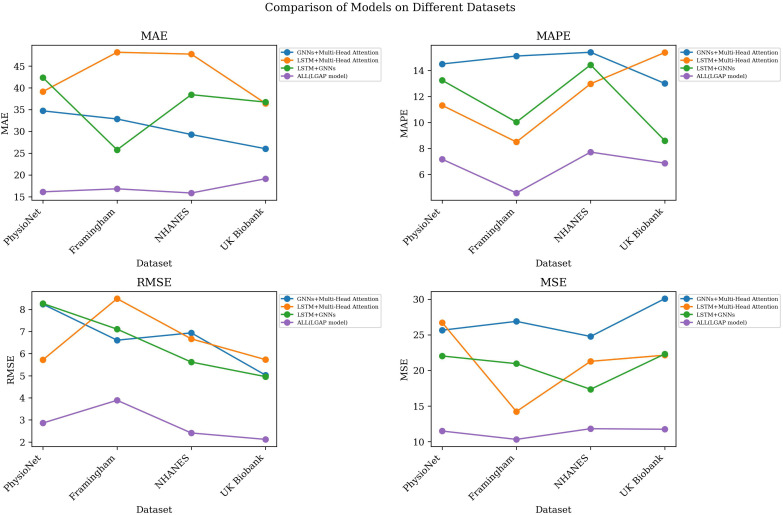
Ablation experiments on the LGAP model.

In order to present the comparison results more intuitively, we visually display the table contents, as shown in **Figure 7**. The figure can provide a clearer understanding of the performance of the LGAP model in the ablation experiment, as well as the impact of different components on the model performance.

Based on the above experimental results, the ablation experimental results further verify the importance of each component in the LGAP model. LSTM’s processing and feature extraction of time series data, GNNs’ mining of relationships between patients, and Multi-Head Attention’s fusion of different modal data all play a crucial role in model performance.

**Figure 8:**
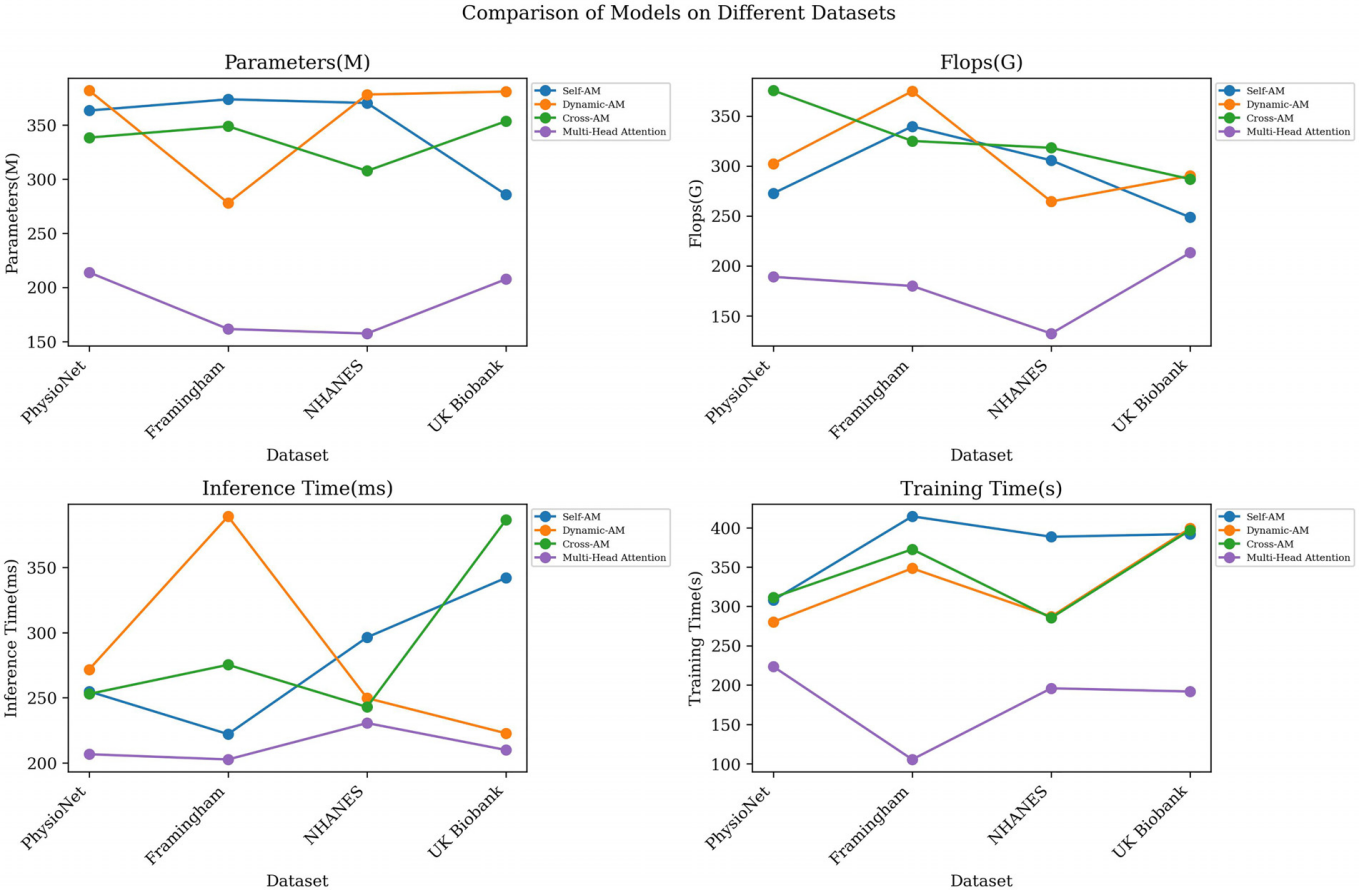
Comparative experiments on the Multi-Head Attention.

Focusing on optimizing the attention mechanism, we compared the differences in model performance among four different attention mechanisms: Self-AM, Dynamic-AM, Cross-AM and Multi-Head Attention. **Table 5** outlines the outcomes from our comparative analysis, detailing key performance indicators such as model parameters, computational complexity, and the times for inference and training across various datasets. **Figure 8** intuitively displays these experimental results through visualization.

**Table 5:** Comparative experiments on the Multi-Head Attention using different datasets.

Model	Datasets															
	PhysioNet				Framingham Heart Study Dataset				NHANES				UK Biobank			
	Parameters(M)	Flops(G)	Inference Time(ms)	Trainning Time(s)	Parameters(M)	Flops(G)	Inference Time(ms)	Trainning Time(s)	Parameters(M)	Flops(G)	Inference Time(ms)	Trainning Time(s)	Parameters(M)	Flops(G)	Inference Time(ms)	Trainning Time(s)
Self-AM	363.55	272.6	254.94	307.88	373.91	339.63	222.1	414.49	370.53	305.63	296.46	388.54	285.73	248.6	342.18	392.02
Dynamic-AM	382.12	302.27	271.75	280.33	278.06	374.85	389.35	348.41	378.35	264.44	249.78	287.08	381.11	290.21	222.75	399.43
Cross-AM	338.51	375.61	252.97	311.34	348.98	325.06	275.36	372.62	307.72	318.21	242.93	285.43	353.69	286.83	386.7	397.14
Multi-Head Attention	213.87	189.05	206.68	223.56	161.51	179.93	202.68	105.43	157.33	132.33	230.62	195.91	207.6	213.23	209.93	191.8

First, compare the differences in the number of model parameters and computational complexity of different attention mechanisms. We can observe that the three attention mechanisms of Self-AM, Dynamic-AM and Cross-AM have relatively high parameter amounts and computational complexity, while Multi-Head Attention has a lower parameter amount and computational complexity. For example, on the PhysioNet data set, the parameter amount of Self-AM is 363.55M, while Multi-Head Attention is only 213.87M, which reflects the advantages of Multi-Head Attention in saving storage space and computing resources.

Secondly, compare the performance of different attention mechanisms in terms of inference time and training time. In terms of inference time, Multi-Head Attention generally exhibits faster inference speed, which can be seen from the inference time data in **Table 4**. For example, on the Framingham Heart Study Dataset, the inference time of Multi-Head Attention is 105.43ms, which is significantly lower than other attention mechanisms. In terms of training time, the differences between various attention mechanisms are not obvious, but the general trend is that Multi-Head Attention usually has shorter training time, which helps to improve the training efficiency of the model.

Finally, compare the differences in model prediction accuracy between different attention mechanisms. By comparing the performance metrics of each model on different data sets, we can evaluate their performance in terms of model prediction accuracy. Generally speaking, Multi-Head Attention shows better performance on indicators such as MAE, MAPE, RMSE, and MSE, which shows that Multi-Head Attention has certain advantages in improving model prediction accuracy.

Based on the above experimental results, it is shown that our choice of Multi-Head Attention as the optimization mechanism of the model is very suitable. It can coordinate other components of the model and greatly improve the performance of the model.

## 5 Conclusion and discussion

In the research of this article, deep learning technology is mainly used to solve the challenges in cardiovascular disease prediction. To address this problem, we established the LGAP model, which combines key components such as Long Short-Term Memory Network (LSTM), Graph Neural Networks (GNNs), and Multi-Head Attention. The integration of this component enables us to extract key features from patients’ time-series data and dig deeper into behavioral patterns and correlations between patients. Experimental results show that the LGAP model has significant advantages in cardiovascular disease prediction. In ablation experiments and comparative analysis, we found that the LGAP model performed well in terms of prediction accuracy and performance stability. Taken together, the model in this article provides an effective solution for cardiovascular disease prediction.

However, although the LGAP model has shown many advantages in cardiovascular disease prediction, it also has some shortcomings. For example, when processing large-scale data, the model may face a certain computational burden, which may affect the real-time performance and efficiency of the model. In order to overcome this challenge, the structure and algorithm of the LGAP model need to be further optimized to improve its processing capabilities on large-scale data sets, including optimizing the utilization of computing resources and improving the parallel computing capabilities of the model. In addition, for certain specific types of data, such as sparse data or data with complex nonlinear relationships, the prediction effect of the LGAP model may be limited, which requires more data and experimental verification for further exploration and improvement.

Future research will further deepen the application and optimization of the LGAP model to achieve more precise and personalized health management and prevention work. In addition, it will be combined with advanced technologies in other fields, such as bioinformatics, medical image processing, etc., to further expand the application scenarios and functions of the LGAP model, so as to better provide support and services for health management and medical prevention.

## Data Availability

The original contributions presented in the study are included in the article/supplementary material. Further inquiries can be directed to the corresponding author.
